# Effect of different physical training modalities on peak oxygen consumptions in post-acute myocardial infarction patients: systematic review and meta-analysis

**DOI:** 10.1590/1677-5449.210056

**Published:** 2021-08-06

**Authors:** Gabriela Bourscheid, Karin Raquel Just, Rochelle Rocha Costa, Thalia Petry, Luiz Cláudio Danzmann, Adamastor Humberto Pereira, Alexandre Araújo Pereira, Leandro Tolfo Franzoni, Eduardo Lima Garcia

**Affiliations:** 1 Faculdade Cenecista de Santo Ângelo, Santo Ângelo, RS, Brasil.; 2 Universidade Federal do Rio Grande do Sul – UFRGS, Porto Alegre, RS, Brasil.; 3 Faculdade SOGIPA, Porto Alegre, RS, Brasil.; 4 Universidade Federal de Santa Maria – UFSM, Santa Maria, RS, Brasil.; 5 Universidade Luterana do Brasil – ULBRA, Canoas, RS, Brasil.; 6 Hospital de Clínicas de Porto Alegre – HCPA/UFRGS, Porto Alegre, RS, Brasil.

**Keywords:** physical exercise, acute myocardial infarction, cardiovascular rehabilitation, coronary artery disease, ischemic cardiopathy

## Abstract

Physical training can increase peak oxygen uptake (VO2peak) in people who have suffered acute myocardial infarction (AMI). However, there is still a gap in the literature in relation to the effectiveness of different types of interventions. Therefore, the aim of the present study was to evaluate the effects of different physical training modalities on VO2peak in post-AMI patients. The following databases were used: PubMed (MEDLINE), Cochrane Library, Scopus, and Pedro. Studies that evaluated aerobic exercise, strength exercise, or combined exercise were included. Six studies met eligibility criteria. Aerobic exercise increased VO2peak by 6.07 ml.kg^-1^.min^-1^ when compared to the control group (CG) (p = 0.013). The comparison between combined exercise and control group detected a difference of 1.84 ml.kg^-1^.min^-1^, but this was not significant (p = 0.312). We therefore conclude that aerobic exercise is the only modality that is effective for increasing VO2peak compared to a control group.

## INTRODUCTION

Mortality due to cardiovascular diseases (CVDs) accounts for around 70% of deaths globally, at more than 38 million deaths per year.^1^ In Brazil, the CVD mortality rate has attained 30%.^2^ From 2008 to 2016, the Brazilian National Health Service (SUS - Sistema Único de Saúde) performed 2,548,944 procedures involving ischemic heart diseases, ranging from clinical treatment for acute myocardial infarction (AMI) to myocardial revascularization surgery.^3^ In 2018, the SUS spent around R$ 3,700,000.00 on procedures involving myocardial ischemia.^3^ It is estimated that AMI mortality was 56% in 2017.^4^

Acute myocardial infarction is clearly related to atherosclerotic disease load,^5^ but is even more directly linked to acute occlusive formation of thrombosis on coronary atheromatous plaques, occluding the vessel lumen and causing myocardial necrosis.^6^^,^^7^ The degree of irreversibility of atherosclerosis is directly associated with advanced lesions, such as, for example, fibroatheromas.^8^ Necrosis formed by a lipid-rich core is caused by degradation of the extracellular matrix, death of smooth muscle cells, and apoptosis of foam cells, provoking build-up of lipids.^9^ Finally, fibroatheromas cause arterial calcification, composing part of the occlusive plaque, which is defined by presence of arterial thrombus.^10^

Post-infarction myocardial dysfunction is the predominant factor in impairment of patients’ functional capacity. Changes to the cardiac muscle’s capacity for contractility makes it unable to increase heart rate and arterial blood pressure at low levels of physical effort, reducing the double product and generating a low ischemic threshold. Cardiopulmonary exercise testing is considered the gold standard for assessment of functional capacity, in terms of peak oxygen consumption (VO2peak).^11^^,^^12^ Many different studies have demonstrated that VO2peak is an independent predictor of mortality in people who have had AMI.^13^^-^15 Conversely, a 1 mL.kg^-1^.min^-1^ increase in VO2peak is directly associated with a 10% reduction in risk of CVD mortality.^16^^,^^17^

Physical exercise is an extremely important non-pharmaceutical tool for treating AMI, both for preventing risk factors and for increasing VO2peak.^18^ Aerobic exercise is the most recommended modality in current cardiac rehabilitation guidelines,^19^^-^21 because it is the simplest to perform outside of a hospital environment and does not require equipment.^22^ With regard to efficacy, aerobic exercise appears to deliver similar results in terms of VO2peak, compared to other methods, such as, for example, combined exercise (aerobic + strength exercises in the same session),^23^ and superior results to strength exercises.^24^ However, there is a gap in the literature in relation to comparisons of the efficacy of different types of physical training for improving VO2peak in people who have had AMI. In view of this, the objective of the present study was to conduct a meta-analysis comparing the effects of different types of physical training on the VO2peak of post-AMI patients.

## METHODS

### Study characteristics

The study design is a systematic review with meta-analysis. Recommendations proposed by the Cochrane Collaboration^25^ and the Preferred Reporting Items for Systematic Review and Meta-analyses (PRISMA) statement were followed.^26^ The review was registered in advance with the International Prospective Register of Systematic Reviews (PROSPERO) (CRD42020182666).

### Eligibility criteria

The review included randomized clinical trials assessing the effect of physical exercise on VO2peak in patients who had had AMI, with no limits on age or sex. Additionally, studies were also included that subjected patients to myocardial/endovascular revascularization surgery or conservative treatment with antithrombotic medications to treat the ischemic condition.

Physical exercise modalities were restricted to three types: aerobic exercise, strength exercises, and combined exercise, with no restriction in relation to the type of exercise, instruments, intensity, session duration, weekly frequency, volume, or rest interval. The minimum intervention duration was set at 6 weeks, considered the minimum time necessary for a positive effect on VO2peak in post-AMI patients, and exercise should be supervised.

Additionally, studies should report comparisons of results with groups of patients who did not perform exercise (control group) or performed the exercise recommended by guidelines (literature standard control group –moderate intensity aerobic exercise). Only studies that did not report significant differences between the groups before the follow-up period started were included. No publication date limits were imposed and studies published in English and Portuguese were included in the analysis.

### Exclusion criteria

Studies comparing surgical techniques with non-surgical techniques were excluded from this systematic review. Observational studies and studies that did not report sufficient data for data extraction were also excluded.

## DATA COLLECTION PROCEDURES

### Search strategy

Initially, articles were filtered using EndNote software to exclude duplicates. Titles and abstracts were then independently analyzed against the eligibility criteria by two experienced assessors. After article selection, the full texts of studies that met the eligibility criteria were analyzed. No publication date filter was applied, in order to include a larger range of studies.

We used the following electronic databases: PubMed (MEDLINE – US National Library of Medicine), Cochrane Library, Scopus, and Pedro (Physiotherapy Evidence Database). Manual searches of the reference lists of studies included in the review were also conducted. No filter was set to select randomized clinical trials, since the decision had been made to initially include more studies, to widen the scope of the literature on the subject reviewed. Selection on the basis of study design was performed manually. Abstracts and extended abstracts published in conference annals, dissertations, theses, and studies not yet published in journals (pre-print) were not included.

A PICO question was constructed to define the search string and guide the study selection strategy:

Population: patients with acute myocardial infarction;

Intervention: physical exercise (aerobic, strength, or combined);

Comparison: control group, placebo, or aerobic exercise;

Outcomes: peak oxygen consumption.

The search string used for PubMed was as follows:

Population: “Myocardial Infarction”[Mesh] OR “Infarction, Myocardial” OR “Infarcts, Myocardial” OR “Myocardial Infarcts” OR “Cardiovascular Stroke” OR “Cardiovascular Strokes” OR “Stroke, Cardiovascular” OR “Strokes, Cardiovascular” OR “Myocardial Infarct” OR “Infarct, Myocardial” OR “Infarcts, Myocardial” OR “Myocardial Infarcts” OR “Heart Attack” OR “Heart Attacks”

Intervention: “Exercise”[Mesh] OR “Exercises” OR “Physical Activity” OR “Activities, Physical” OR “Activity, Physical” OR “Physical Activities” OR “Exercise, Physical” OR “Exercises, Physical” OR “Physical Exercise” OR “Physical Exercises” OR “Acute Exercise” OR “Acute Exercises” OR “Exercise, Acute” OR “Exercises, Acute” OR “Exercise, Isometric” OR “Exercises, Isometric” OR “Isometric Exercises” OR “Isometric Exercise” OR “Exercise, Aerobic” OR “Aerobic Exercise” OR “Aerobic Exercises” OR “Exercises, Aerobic” OR “Exercise Training” OR “Exercise Trainings” OR “Training, Exercise” OR “Trainings, Exercise” OR “Resistance Training”[Mesh] OR “Training, Resistance” OR “Strength Training” OR “Training, Strength” OR “Weight-Lifting Strengthening Program” OR “Strengthening Program, Weight-Lifting” OR “Strengthening Programs, Weight-Lifting” OR “Weight Lifting Strengthening Program” OR “Weight-Lifting Strengthening Programs” OR “Weight-Lifting Exercise Program” OR “Exercise Program, Weight-Lifting” OR “Exercise Programs, Weight-Lifting” OR “Weight Lifting Exercise Program” OR “Weight-Lifting Exercise Programs” OR “Weight-Bearing Strengthening Program” OR “Weight-Bearing Strengthening Program” OR “Strengthening Program, Weight-Bearing” OR “Strengthening Programs, Weight-Bearing” OR “Weight Bearing Strengthening Program” OR “Weight-Bearing Strengthening Programs” OR “Weight-Bearing Exercise Program” OR “Exercise Program, Weight-Bearing” OR “Exercise Programs, Weight-Bearing” OR “Weight Bearing Exercise Program” OR “Weight-Bearing Exercise Programs”.

We decided not to include outcomes in the search string, in order to include a wide range of literature on the central subject, including only population and intervention.

In the other databases we only used the MeSH terms “Myocardial Infarction”, “Exercise”, and “Resistance Training”, since it is unnecessary to include all the other terms for Cochrane Library, Pedro, or SciELO.

## SELECTION OF STUDIES AND DATA EXTRACTION

Two independent reviewers (GB and KRJ) assessed the titles and abstracts of all the articles identified by the search strategy. The full texts of all articles selected and any with respect to which there was any doubt were then read by the same two independent assessors, applying the criteria for inclusion and exclusion of the studies. Any discrepancies between these two assessors’ decisions were resolved by consensus. In cases of disagreement or doubt, a third evaluator (LTF) was available to decide on inclusion or exclusion of the study in question.

The same independent assessors performed data extraction. A standardized form was constructed, indicating the information that should be extracted, including, for example, sample characteristics, relevant clinical information, such as time since AMI, medications used, and complete and detailed descriptions of the interventions administered. The following data were extracted: year of study, sample size, sex, number of men, number of women, mean age, standard deviation of age, mean body mass, standard deviation of body mass, mean body mass index (BMI), standard deviation of BMI, characteristics of groups, time since AMI, type of surgery, medications, type of training, weekly frequency, duration of follow-up, progression, series, repetitions, intensity, interval, and volume. Additionally, data were extracted on the primary outcome of the study and VO2peak (mean and standard deviation for pre and post-intervention periods).

## ASSESSMENT OF METHODOLOGICAL QUALITY (RISK OF BIAS)

Cochrane Collaboration recommendations were followed with relation to evaluation of methodological quality,^25^ extracting data on: generation of randomization sequence, allocation concealment, blinding of patient and therapist, blinding of outcome examiners, description of losses and exclusions, and incomplete outcome data. Two independent reviewers participated in this phase of the assessment (ELG and LTF), and for each criterion the studies were classified as high risk (if the criterion was not present), low risk (if the criterion was present), or unclear risk (if the criterion was not reported).

## ANALYSIS OF THE DATA

Results are expressed as standardized mean difference in absolute values between interventions with the 95% confidence interval (95%CI). Statistical heterogeneity of the effects of interventions between studies was assessed by the Cochran Q test and the I*^2^* test of inconsistency, for which values exceeding 50% indicate high heterogeneity.^27^ The random effects model was employed. The meta-analysis analyzed values for comparisons of VO2peak, expressed in mL.kg^-^.^1^ min^-1^, for aerobic exercise vs. control and combined exercise vs. control. Results with α ≤ 0.05 were considered statistically significant. All analyses were conducted using Comprehensive Meta-Analysis version 2.0 (Englewood, New Jersey, USA).

## RESULTS

**Figure 1:**
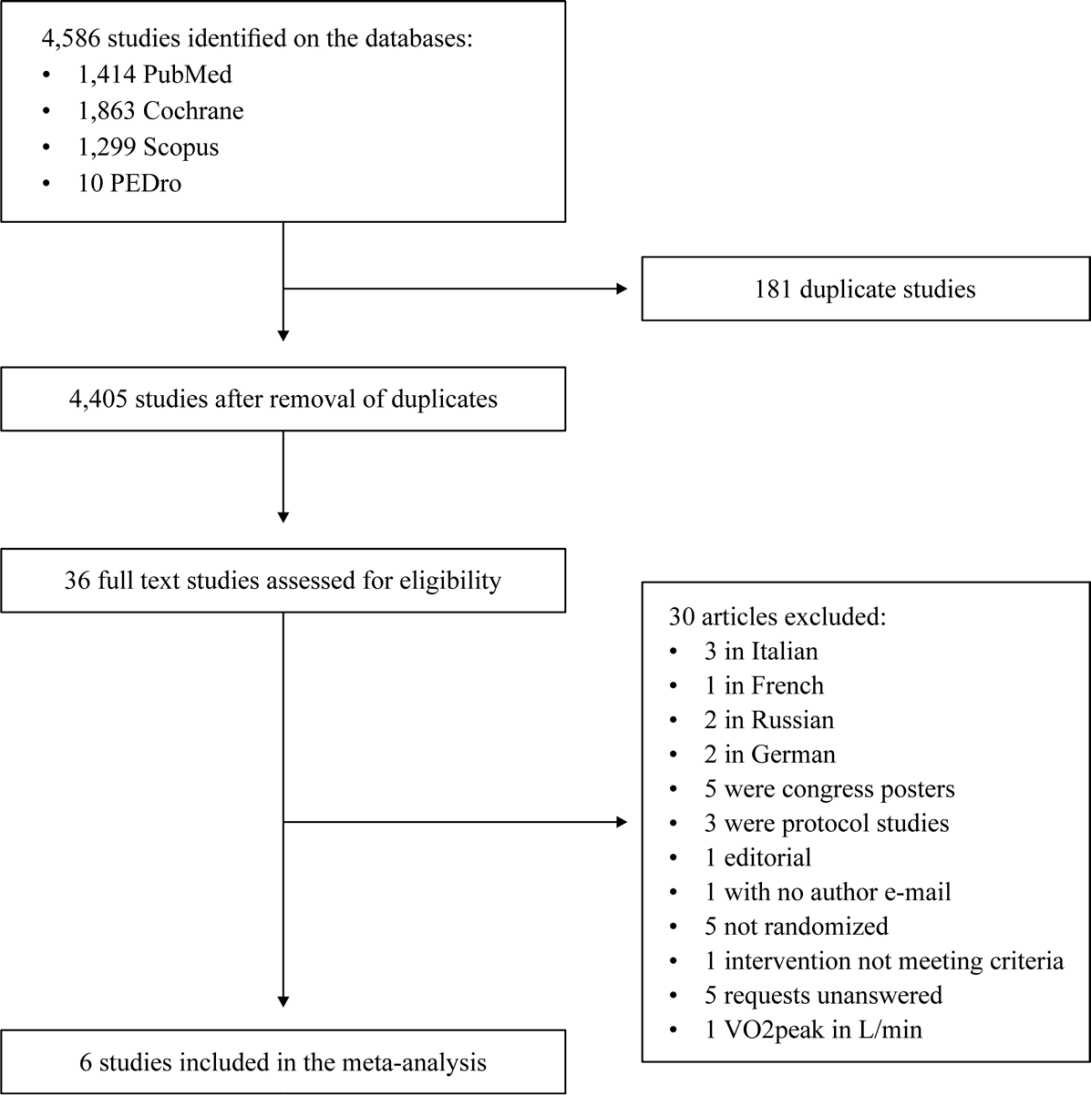
Study flow diagram illustrating all steps in the systematic review and meta-analysis.

Eight of the 4,586 studies identified met the inclusion criteria (Figure 1). However, the VO2peak data from one study were reported in L.min^-1^ and another was not randomized.^28^^,^^29^ After the authors of the first of these studies were contacted, they stated that they did not have data in mL.kg^-1^.min^-1^, which is the standardized measure for expressing this variable. As a result, six studies were included in the quantitative analysis.^30^^-^35 Two of these were included twice^30^^,^^32^ because they met eligibility criteria for two comparisons between groups: aerobic exercise of moderate intensity vs. control and aerobic exercise of high intensity vs. control. Additionally, two studies met the eligibility criteria for comparison of combined exercise vs. control (aerobic training).

Overall, 361 participants were included in the meta-analysis. Of these, 155 were allocated to an aerobic exercise group (AEG), 35 to a combined exercise group (CEG), and 171 to a control group (CG). Fifty per cent of the studies analyzed male patients only,^30^^,^^33^^,^^34^ 33% analyzed people of both sexes, and just one study (17%) did not report the sex of its sample. The total number of women was 35 (9.7%).

Two studies (33%) studied samples comprising a sedentary group with a history of obesity.^30^^,^^33^ Additionally, 50% exhibited risk factors such as diabetes mellitus, systemic arterial hypertension, dyslipidemia, and history of smoking.^31^^,^^33^^-^35 Aerobic exercise was the most prevalent modality among the six studies (66%) and two of the four studies of aerobic exercise were included twice, because they investigated different exercise intensities. Moderate intensity was used in three of these four studies (75%),^30^^,^^32^^,^^33^ and high intensity was used in three studies (75%).^30^^,^^32^^,^^35^ Combined exercise was used in two studies (34%), both of which used high intensity aerobic exercise as part of combined exercise.^31^^,^^34^

Mean duration of the intervention programs was 10.33 ± 2.66 weeks, mean weekly frequency was 3.17 ± 0.98, and mean duration of sessions was 45.83 ± 9.52 minutes. Sixty-six percent of the six studies included reported mean time since participants had suffered AMI (22 ± 21 weeks). Table 1 summarizes the main characteristics of the studies included. Table 2 lists aspects related to risk of bias.

**Table 1:** Characteristics of studies included in data extraction.

**Study**	**Participants**	**Number of participants**	**Medications**	**Time since AMI**	**Mean Age (years)**	**Intervention characteristics**	**Comparison characteristics**	**Progression**	**Series**	**Repetitions**	**Intensity**	**Frequency**	**Follow up**	**Duration**
				**(weeks)**										
Benetti et al., 2010	Obese and sedentary patients	MAG: 29	X	MAG: 36	57.7 ± 6.1	Moderate intensity aerobic training and high intensity interval training	Instructed to maintain normal routine	No	X	X	MAG: 75% of Max HR	5	12	60 min
		HIAG: 29		HIAG: 28							HIAG: 85% of Max HR			
		CG: 29		CG: 36										
Moholdt et al., 2011	Patients with history of smoking	HIAG: 30	Beta blockers,	12	HIAG: 56.7 ± 10.4	High intensity aerobic interval training, on a treadmill, at home, and in hospital	Moderate intensity aerobic training, with climbing staircases and squats	No	HIAG: 4	HIAG: 4	HIAG: 85-95% of Max HR	3	12	51 min
		CG: 59	ASA,		CG:						CG: X			
			ACEI,		57.5 ± 9.3									
			statins,											
			calcium antagonists											
			
Oliveira et al., 2014	Diabetic, hypertensive, dyslipidemic, obese, sedentary, and smoking patients	MAG: 47	Antiplatelet drugs, beta blockers, CCB, ACEI, antihyperlipidemics, diuretics, nitrate angiotensin II receptor blockers	4	MAG: 54.8 ± 10.6	Moderate intensity aerobic training, on a treadmill and a cycle ergometer	Instructed to maintain normal care	No	X	X	70-85% of Max HR	3	8	50 min
		CG: 37			CG:									
					58.6 ± 10.7									
**Study**	**Participants**	**Number of participants**	**Medications**	**Time since AMI**	**Mean Age (years)**	**Intervention characteristics**	**Comparison characteristics**	**Progression**	**Series**	**Repetitions**	**Intensity**	**Frequency**	**Follow up**	**Duration**
				**(weeks)**										
Santi et al., 2018	X	MAG: 10	X	X	55.1 ± 8.9		Normal care	No	HIAG: 4	HIAG: 4	MAG: 60-70% of Max HR	3	12	MAG: 40 min
		HIAG: 10				Moderate intensity aerobic training and high intensity interval training					HIAG: 85-95% of Max HR			HIAG: 28 min
		CG: 10												
Khalid et al., 2019	Diabetic and hypertensive patients	CTG: 26	X	X	CTG: 57.23 ± 9.75	Aerobic interval training + strength training	Aerobic interval training	10 repetitions initially, increasing to 12 repetitions of strength training	X	X	CTG: aerobic – 60-85% Max HR	3	6	CTG: 40 min
		CG: 26			CG:						Strength - X			CG: 65 min
					55.77 ± 10.45						CG: 60-85% Max HR			
Traschel et al., 2019	Diabetic, dyslipidemic, and hypertensive, and smoking patients	CTG: 9	Aspirin, beta blockers, CCB, DPA, antihyperlipidemic agents, RAASI	6	CTG:	High intensity aerobic interval training + strength training	Moderate intensity aerobic training	No	Aerobic:	Strength:	CTG: aerobic – 100% of peak workload (BORG 15)	2	12	CTG: 40 min
		CG: 10			60 ± 10				2 to 3	15 to 20	Strength – BORG 15			CG:
					CG:				Strength: 1		CG: BORG 12 – 14			30 – 60 min
					57 ± 13									

AMI = acute myocardial infarction; MAG = moderate aerobics group; HIAG= high intensity aerobics group; CTG = combined training group; CG = control group; HR= heart rate; Max HR = maximum heart rate; BORG = subjective perceived exertion scale; ASA = acetylsalicylic acid; ACEI= angiotensin-converting enzyme inhibitors; RAASI = renin-angiotensin-aldosterone system inhibitors ; CCB= calcium channel blockers; DPA= double platelet antiaggregation.

**Table 2:** Extraction of risk of bias.

**Study**	**Generation of randomization sequence**	**Allocation concealment**	**Blinding of patient and therapist**	**Blinding of outcome assessors**	**Description of losses and exclusions**	**Incomplete outcome data**
Benetti et al., 2010	Low	*Unclear*	*Unclear*	*Unclear*	Low	*Unclear*
Moholdt et al., 2011	Low	Low	Low	Low	Low	Low
Oliveira et al., 2014	Low	Low	High	Low	Low	Low
Santi et al., 2018	Low	*Unclear*	*Unclear*	*Unclear*	*Unclear*	*Unclear*
Khalid et al., 2019	Low	*Unclear*	High	Low	Low	High
Traschel et al., 2019	Low	*Unclear*	High	Low	High	*Unclear*

Low = criterion present, considered low risk of bias; High = criterion not present; Unclear = not clear whether criterion present or not.

## EFFECTS OF THE INTERVENTIONS

### Aerobic exercise *vs.* Control (with and without intervention)

**Figure 2:**
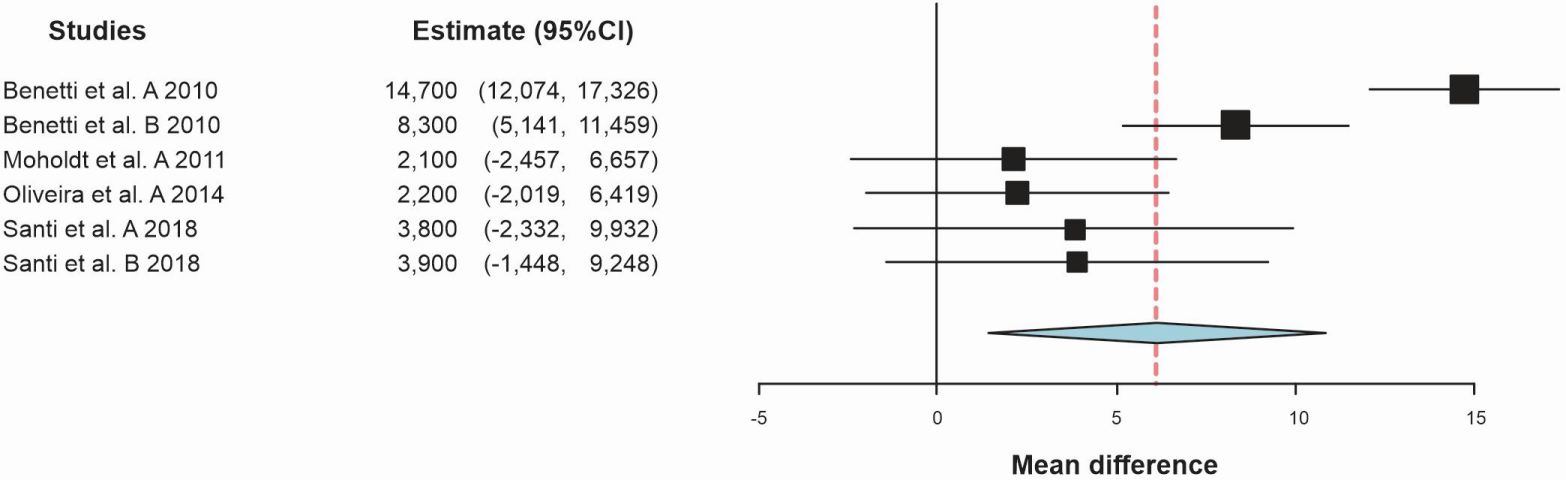
Standardized mean differences in peak oxygen consumption (mL.kg^-1^.min^-^ **)** observed between aerobic exercise group and control group.

**Note:** Solid squares = estimates from specific studies; Solid diamond = combined estimate from random effects meta-analyses; Standardized difference; Confidence interval.

A total of 290 participants were assessed (Figure 2), 155 in the AEG and 135 in the CG. Aerobic exercise was associated with a mean increase of 6.07 mL.kg^-1^.min^-1^ (95%CI 1.27 to 10.86; *I^2^*: 88%) when compared to CG (p = 0.013).

### Combined Exercise *vs.* Control (without intervention)

**Figure 3:**
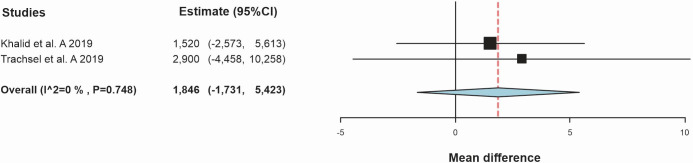
Standardized mean differences in peak oxygen consumption (mL.kg^-1^.min^-^ **)** observed between combined exercise group and control group.

**Note:** Solid squares = estimates from specific studies; Solid diamond = combined estimate from random effects meta-analyses; Standardized difference; Confidence interval.

Seventy-one participants were assessed (Figure 3), 35 in the CEG and 36 in the CG. Combined exercise did not result in a statistically significant difference when compared with the CG. Combined exercise did, nevertheless, result in a mean increase of 1.84 mL.kg^-1^.min^-1^ (95%CI -1.73 to 5.42; *I^2^*: 0%) when compared to the CG (p = 0.312).

## DISCUSSION

This study conducted a meta-analysis to investigate the effects of different types of physical training on the VO2peak of individuals who had suffered an AMI. The main finding is that aerobic exercise was associated with a mean VO2peak increase of 6 mL.kg^-1^.min^-1^ compared with the CG, which was a statistically significant difference (p = 0.013).

Although this systematic review investigated the effects of different types of physical training, it is important to stress that very few studies have investigated the effects of combined exercise on the VO2peak of individuals who have suffered AMI^31^^,^^34^ and that no studies have investigated the effects of strength exercises on VO2peak. In contrast, the modality most investigated in these studies was aerobic exercise (66%).^30^^,^^32^^,^^33^^,^^35^ Moreover, high intensity aerobic exercise was used as the CG in the two studies that investigated combined exercise. Our findings therefore corroborate the literature that indicates aerobic exercise as the most used modality for rehabilitation of patients who have suffered an AMI.^19^^,^^20^

A total of 361 participants were included in this meta-analysis, 296 of whom were men, which underscores the prevalence of AMI among men.^4^ It is therefore important to emphasize that one of the risk factors for development of this disease is sex, in addition to age group.^5^ The main physiological protective factor in women is estrogen, a hormone directly related to cardiovascular protection.^36^ In addition to sex, AMI affects people from 40-45 years of age onwards. At this age, women’s estrogen production is maintained. The highest incidence of AMI among women is after menopause, when there is a considerable drop in estrogen production.^36^

It has been shown that aerobic exercise is effective for reducing risk factors and for increasing functional capacity in middle-aged or elderly people with a range of CVDs.^22^^,^^37^ Physical training programs including aerobic exercise are important to provoke positive adaptations in the VO2peak of people who have suffered an AMI. However, it is essential to manage the intensity of exercise to achieve the desired results during an intervention.^38^ High intensity aerobic exercise appears to deliver the greatest increase in VO2peak (14.7 mL.kg^-1^.min^-1^).^30^ High intensity (at 85-95% of VO2peak) is associated with good adherence to intervention programs using physical exercises and is also linked with increased oxidative capacity of the muscles and glucose transport capacity, improving insulin sensitivity and glycemic control as a consequence.^39^ The principal mechanism of adaptation in response to high intensity exercise is related to peroxisome proliferator-activated receptor gamma coactivator 1-alpha, the most important regulator of mitochondrial biogenesis in muscle.^40^

The regulator mechanism of moderate intensity aerobic exercise appears to be similar. The central aspect is mitochondrial biogenesis, an important cellular organelle responsible for the oxidative activity of muscles.^41^^,^^42^ Apparently, 6 weeks of moderate intensity aerobic exercise are needed to provoke increases in both the size and the number of mitochondria, increasing the capacity for resynthesis of adenosine triphosphate. This corroborates one of the inclusion criteria for the present study, since a minimum of 6 weeks of intervention was established.^43^ Moreover, aerobic exercise in general increases arteriovenous oxygen difference, which is directly related to increased VO2peak, via greater peripheral oxygen supply, as a result of production of catecholamines and greater bioavailability of nitric oxide.^44^^,^^45^ Cardiac function also exhibits improved performance after an intervention with aerobic exercise, with increased diastolic filling and a concomitant combination of increased preload and optimized myocardium relaxation.^46^ The improved cardiac function will provoke important benefits for reduction of diastole duration and more efficient diastolic filling.^46^

From a clinical point of view, the main finding of this study corroborates the literature on the role of rehabilitation with aerobic exercises of patients who have suffered AMI, utilizing moderate intensity as a preference,^47^ since the several guidelines have recommendation strength I with evidence level A; i.e., this modality is highly recommended for several different CVDs.^19^^-^21 The mean increase of 6 mL.kg^-1^.min^-1^ in VO2peak – the main finding of this study – demonstrates that physical exercise plays a fundamental role in reducing the risk of mortality from CVDs, since an increase of one unit of VO2peak represents a 10% reduction in the risk of CVD mortality.^16^^,^^17^ Furthermore, the 6 mL.kg^-1^.min^-1^ difference could increase patients’ autonomy to perform their daily activities, since this difference can constitute a change in functional class, whether New York Heart Association (NYHA) or the Weber class, whereby, for example, a patient at NYHA class III and Weber class C could attain NYHA class I and Weber class A.^47^^,^^48^ The finding of the present study is therefore extremely relevant to cardiovascular rehabilitation.

Although combined exercise did not exhibit significant differences compared to the CG (p = 0.312), it must be emphasized that only two studies using this type of intervention were included, which could be extremely relevant to not detecting significance. The mean difference in increase in VO2peak for combined exercise compared with the CG was 1.84 mL.kg^-1^.min^-1^. The combination of aerobic exercise and strength exercises in the same session appears to be a promising strategy – since in addition to promoting improved VO2peak, several studies point to the importance of combined exercise for improving neuromuscular aspects related to balance and muscle strength, primarily as a result of strength exercises.^23^^,^^29^ Indeed, strength exercises provoke increases in the size of muscle fibers, with a consequent increase in the number of mitochondria, facilitating oxidative muscle activity.^49^ This is why it is important that future studies should investigate different physical training programs in relation to different outcomes among people who have suffered AMI.

We cannot fail to highlight the extensive search of the literature for scientific articles with high methodological quality and the best available evidence level. More than 4,000 studies were selected for systematic review and their titles and abstracts were read. To our knowledge this is the first meta-analysis to investigate the effects of different physical training modalities on the VO2peak of post-AMI patients.

## CONCLUSIONS

Our data demonstrated that those participants who trained with the aerobic exercise modality obtained a significant increase in VO2peak. Additionally, this was the modality most used in the studies included in the meta-analysis. Combined exercise was not associated with a significant increase in VO2peak, but its use in cardiovascular rehabilitation programs is extremely important, since patients who suffer AMI do not only have reduced functional capacity, but also lose muscle strength and balance. It is therefore essential to encourage physical training programs for the post-AMI population, with appropriate control of exercise intensity and volume.
