# Supplementary material for: Effect of different physical training modalities on peak oxygen consumptions in post-acute myocardial infarction patients: systematic review and meta-analysis
Source: J Vasc Bras. 2021 Aug 6;20:e20210056. doi: 10.1590/1677-5449.210056 (PMC8354336; doi:10.1590/1677-5449.210056)
Supplement: Translation

# Translation: Efeito de diferentes modalidades de treinamento físico no consumo de oxigênio de pico em pacientes pós-infarto agudo do miocárdio: uma revisão sistemática e metanálise

**Authors:** Gabriela Bourscheid, Karin Raquel Just, Rochelle Rocha Costa, Thalia Petry, Luiz Cláudio Danzmann, Adamastor Humberto Pereira, Alexandre Araújo Pereira, Leandro Tolfo Franzoni, Eduardo Lima Garcia

**Affiliations:** 1 Faculdade Cenecista de Santo Ângelo, Santo Ângelo, RS, Brasil.; 2 Universidade Federal do Rio Grande do Sul – UFRGS, Porto Alegre, RS, Brasil.; 3 Faculdade SOGIPA, Porto Alegre, RS, Brasil.; 4 Universidade Federal de Santa Maria – UFSM, Santa Maria, RS, Brasil.; 5 Universidade Luterana do Brasil – ULBRA, Canoas, RS, Brasil.; 6 Hospital de Clínicas de Porto Alegre – HCPA/UFRGS, Porto Alegre, RS, Brasil.

**Keywords:** exercício físico, infarto agudo do miocárdio, reabilitação cardiovascular, doença da artéria coronariana, cardiopatia isquêmica

## Abstract

O treinamento físico é capaz de aumentar o consumo de oxigênio de pico em indivíduos que sofreram infarto agudo do miocárdio. No entanto, em relação à eficácia de diferentes tipos de intervenções ainda há uma lacuna na literatura. O objetivo do presente estudo foi avaliar os efeitos de diferentes modalidades de treinamento físico no consumo de oxigênio de pico de pacientes pós-infarto agudo do miocárdio. Foram utilizadas as seguintes bases de dados*:* PubMed (MEDLINE), Cochrane Library, Scopus e PEDro. Foram incluídos estudos que avaliassem exercícios aeróbicos, de força ou combinados. Seis estudos preencheram elegibilidade. O exercício aeróbico aumentou 6,07 mL.kg^-1^.min^-1^ quando comparado ao grupo controle (p = 0,013). Na comparação entre exercício combinado e grupo controle, foi observada uma diferença de 1,84 mL.kg^-1^.min^-1^, no entanto, sem significância (p = 0,312). Portanto, concluímos que o exercício aeróbico é a única modalidade que apresenta eficácia para aumentar o consumo de oxigênio de pico em comparação a um grupo controle.

## INTRODUÇÃO

A mortalidade por doenças cardiovasculares (DCVs) representa cerca de 70% das mortes no mundo, chegando a mais de 38 milhões de mortes por ano^1^. No Brasil, a taxa de mortalidade por DCVs chega a 30%^2^. Entre 2008 e 2016, o Sistema Único de Saúde (SUS) brasileiro efetuou 2.548.944 procedimentos envolvendo doenças isquêmicas do coração, variando de tratamento clínico para infarto agudo do miocárdio (IAM) até cirurgia de revascularização do miocárdio^3^. Em 2018, o SUS gastou cerca de R$ 3.700.000,00 com procedimentos envolvendo isquemia miocárdica^3^. Estima-se que a mortalidade por IAM em 2017 foi de 56%^4^.

O IAM está francamente relacionado à carga de doença aterosclerótica^5^, mas sobretudo vinculado à aguda formação trombótica oclusiva sobre uma placa ateromatosa coronariana, a qual gera oclusão da luz do vaso e necrose miocárdica^6^^,^^7^. O grau de irreversibilidade da aterosclerose está diretamente associado a lesões avançadas, como, por exemplo, fibroateromas^8^. A necrose formada por um núcleo rico em lipídeos é ocasionada pela degradação da matriz extracelular, morte de células musculares lisas e apoptose das células espumosas, promovendo acúmulo de lipídeos^9^. Por fim, os fibroateromas causam calcificação arterial, compondo parte da placa de oclusão, definida pela presença de um trombo arterial^10^.

A disfunção miocárdica pós-infarto é o fator preponderante à redução da capacidade funcional do paciente. Alterações na capacidade de contratilidade do músculo cardíaco impossibilitam a elevação da frequência cardíaca e pressão arterial em baixas cargas de esforço físico, reduzem o duplo produto e geram um limiar isquêmico baixo. O teste cardiopulmonar de exercício é considerado padrão-ouro para avaliação da capacidade funcional, por meio do consumo de oxigênio de pico (VO2pico)^11^^,^^12^. Diferentes estudos demonstram que o VO2pico é um preditor independente de mortalidade para indivíduos que sofreram IAM^13^^-^15. Além disso, um aumento de 1 mL.kg^-1^.min^-1^ no VO2pico está diretamente associado a uma redução de 10% no risco de mortalidade por DCVs^16^^,^^17^.

Entre as diferentes formas não medicamentosas para tratar o IAM, o exercício físico é uma ferramenta de extrema importância, tanto na prevenção dos fatores de risco como no aumento do VO2pico^18^. O exercício aeróbico é o mais recomendado nas atuais diretrizes de reabilitação cardíaca^19^^-^21, pois é o mais simples de ser realizado fora do ambiente hospitalar, sem necessidade de equipamentos^22^. Sobre a eficácia, o exercício aeróbico parece apresentar resultados semelhantes em relação ao VO2pico comparado a outros modelos, como, por exemplo, exercício combinado (aeróbico + força na mesma sessão)^23^, e ser superior a exercício de força^24^. No entanto, existe uma lacuna na literatura em relação à comparação da eficácia de diferentes tipos de treinamento físico sobre o VO2pico em indivíduos que sofreram IAM. Diante disso, o objetivo do presente estudo foi conduzir uma metanálise, comparando os efeitos de diferentes tipos de treinamento físico sobre o VO2pico de indivíduos que sofreram IAM.

## MÉTODOS

### Caracterização da pesquisa

O estudo foi caracterizado como revisão sistemática com metanálise. Foram seguidas as recomendações propostas pela Cochrane Collaboration^25^ e pelo Preferred Reporting Items for Systematic Review and Meta-analyses (PRISMA) Statement^26^. A presente revisão foi previamente registrada no International Prospective Register of Systematic Reviews (PROSPERO) (CRD42020182666).

### Critérios de elegibilidade

Foram incluídos ensaios clínicos randomizados, que avaliaram o efeito do exercício físico sobre VO2pico em indivíduos que sofreram IAM, sem restrições de idade e sexo. Além disso, foram incluídos estudos que submetessem os indivíduos à cirurgia de revascularização do miocárdio/endovascular ou tratamento conservador com medicamento antitrombótico para reverter o quadro de isquemia.

Os modelos de exercício físico foram restritos a três tipos: exercício aeróbico, exercício de força e exercício combinado, sem restrição em relação ao tipo de exercício, instrumentos, intensidade, duração da sessão, frequência semanal, volume ou intervalo de descanso. A duração mínima da intervenção foi fixada em 6 semanas, entendida como tempo mínimo necessário para o efeito positivo no VO2pico em pacientes após IAM, e sua realização deveria ter acontecido de modo supervisionado.

Além disso, os estudos deveriam apresentar comparações dos resultados com grupos de indivíduos que não realizaram exercício (grupo controle) ou realizaram o exercício preconizado pelas diretrizes (grupo controle padrão pela literatura – exercício aeróbico de moderada intensidade). Apenas foram incluídos estudos que não relataram diferença significativa entre os grupos antes do início do período de seguimento. Não houve restrições quanto à data de publicação para inclusão dos estudos na revisão, e estudos publicados em inglês e português foram incluídos na análise.

### Critérios de exclusão

Foram excluídos da presente revisão sistemática estudos que comparassem técnicas cirúrgicas com técnicas não cirúrgicas. Além disso, foram excluídos estudos observacionais ou estudos que não apresentassem dados suficientes para a extração dos dados.

## PROCEDIMENTOS PARA COLETA DE DADOS

### Estratégia de busca

Os artigos inicialmente passaram por um filtro realizado com o *software* EndNote, para excluirmos as duplicatas. Após, foram analisados títulos e resumos, com base nos critérios de elegibilidade, por dois avaliadores independentes e com experiência. Depois da seleção dos artigos, foram analisados na íntegra aqueles que preencheram os critérios de elegibilidade. Não foi utilizado filtro para data de publicação, visando abranger maior gama de estudos.

Utilizamos os seguintes bancos de dados eletrônicos: PubMed (MEDLINE – US National Library of Medicine), Cochrane Library, Scopus e PEDro (Physiotherapy Evidence Database). Além disso, foi realizada uma busca manual nas referências dos estudos incluídos na pesquisa. Não foi utilizado filtro para ensaios clínicos randomizados, visto que durante a fase inicial optamos por incluir mais estudos para ampliar a abrangência de literatura sobre o assunto. A seleção em relação ao delineamento do estudo foi realizada de maneira manual. Resumos ou resumos expandidos publicados em conferências, dissertações, teses ou estudos ainda não publicados em periódicos (*pre-print*) não foram incluídos.

A pergunta PICO foi desenvolvida com a finalidade de realizar a busca e orientar a estratégia de seleção dos estudos:

População: pacientes que apresentem infarto agudo do miocárdio;

Intervenção: exercício físico (aeróbico, força ou combinado);

Comparadores: grupo controle, placebo ou exercício aeróbico;

*Outcomes* (desfechos): consumo de oxigênio de pico.

A estratégia de busca utilizada no PubMed foi:

Population: “Myocardial Infarction”[Mesh] OR “Infarction, Myocardial” OR “Infarctions, Myocardial” OR “Myocardial Infarctions” OR “Cardiovascular Stroke” OR “Cardiovascular Strokes” OR “Stroke, Cardiovascular” OR “Strokes, Cardiovascular” OR “Myocardial Infarct” OR “Infarct, Myocardial” OR “Infarcts, Myocardial” OR “Myocardial Infarcts” OR “Heart Attack” OR “Heart Attacks”

Intervention: “Exercise”[Mesh] OR “Exercises” OR “Physical Activity” OR “Activities, Physical” OR “Activity, Physical” OR “Physical Activities” OR “Exercise, Physical” OR “Exercises, Physical” OR “Physical Exercise” OR “Physical Exercises” OR “Acute Exercise” OR “Acute Exercises” OR “Exercise, Acute” OR “Exercises, Acute” OR “Exercise, Isometric” OR “Exercises, Isometric” OR “Isometric Exercises” OR “Isometric Exercise” OR “Exercise, Aerobic” OR “Aerobic Exercise” OR “Aerobic Exercises” OR “Exercises, Aerobic” OR “Exercise Training” OR “Exercise Trainings” OR “Training, Exercise” OR “Trainings, Exercise” OR “Resistance Training”[Mesh] OR “Training, Resistance” OR “Strength Training” OR “Training, Strength” OR “Weight-Lifting Strengthening Program” OR “Strengthening Program, Weight-Lifting” OR “Strengthening Programs, Weight-Lifting” OR “Weight Lifting Strengthening Program” OR “Weight-Lifting Strengthening Programs” OR “Weight-Lifting Exercise Program” OR “Exercise Program, Weight-Lifting” OR “Exercise Programs, Weight-Lifting” OR “Weight Lifting Exercise Program” OR “Weight-Lifting Exercise Programs” OR “Weight-Bearing Strengthening Program” OR “Weight-Bearing Strengthening Program” OR “Strengthening Program, Weight-Bearing” OR “Strengthening Programs, Weight-Bearing” OR “Weight Bearing Strengthening Program” OR “Weight-Bearing Strengthening Programs” OR “Weight-Bearing Exercise Program” OR “Exercise Program, Weight-Bearing” OR “Exercise Programs, Weight-Bearing” OR “Weight Bearing Exercise Program” OR “Weight-Bearing Exercise Programs”.

Optamos em não utilizar os desfechos na estratégia de busca com a finalidade de incluir uma ampla a literatura sobre o assunto central, incluindo apenas população e intervenção.

Nas demais bases de dados utilizamos apenas os termos *MeSH “Myocardial Infarction”*, *“Exercise”* e *“Resistance Training”*, visto que para Cochrane Library, PEDro e SciELO não é necessário inserir todos outros termos.

## SELEÇÃO DOS ESTUDOS E EXTRAÇÃO DOS DADOS

Dois revisores independentes (GB e KRJ) avaliaram títulos e resumos de todos os artigos encontrados por meio da estratégia de busca. Posteriormente, foi feita a leitura completa dos artigos selecionados e daqueles sobre os quais se tinha dúvida pelos mesmos dois revisores independentes, seguindo os critérios que determinam inclusão e exclusão dos estudos. As discordâncias entre os dois avaliadores foram resolvidas por consenso. No caso de discordância ou de dúvida, um terceiro avaliador (LTF) estava disponível para decidir pela inclusão ou exclusão do estudo.

Os mesmos revisores independentes realizaram a extração dos dados. Foi criado um formulário padronizado, indicando quais informações deveriam ser selecionadas, incluindo, por exemplo, dados de caracterização da amostra, informações clínicas relevantes, como tempo de IAM, medicamentos utilizados e a descrição completa e detalhada das intervenções realizadas. Os dados extraídos foram: ano do estudo, tamanho amostral, sexo, número de homens, número de mulheres, média de idade, desvio padrão da idade, massa corporal média, desvio padrão da massa corporal, índice de massa corporal (IMC) médio, desvio padrão do IMC, característica do grupo, tempo de IAM, tipo de cirurgia, medicamentos, tipo de treinamento, frequência semanal, duração do seguimento, progressão, séries, repetições, intensidade, intervalo e volume. Além disso, foram extraídos os dados do desfecho principal do estudo, VO2pico (média e desvio padrão do período pré e pós-intervenção).

## AVALIAÇÃO DA QUALIDADE METODOLÓGICA (RISCO DE VIÉS)

Para dados relacionados à extração da qualidade metodológica seguimos as orientações da Cochrane Collaboration^25^, incluindo: geração da sequência aleatória (randomização), sigilo da alocação, cegamento do paciente e terapeuta, cegamento dos avaliadores dos desfechos, descrição de perdas e exclusões e dados de resultados incompletos. Dois revisores independentes participaram dessa fase de avaliação (ELG e LTF) para cada critério, e os estudos foram classificados como alto risco (caso não apresentasse o critério), baixo risco (caso apresentasse o critério) e risco não claro (caso o critério não fosse reportado).

## ANÁLISE DOS DADOS

Os resultados são apresentados como diferença média padronizada para valores absolutos entre as intervenções com intervalo de confiança de 95% (IC95%). Heterogeneidade estatística dos efeitos das intervenções entre os estudos foi avaliada pelo teste Q de Cochran e teste de inconsistência *I^2^*, sendo que valores acima 50% indicam alta heterogeneidade^27^. O modelo de efeitos aleatórios foi aplicado. A metanálise compreendeu valores de comparação de VO2pico, expressos em mL.kg^-1^.min^-1^, entre exercício aeróbico *versus* controle e exercício combinado *versus* controle. Valores de α ≤ 0,05 foram considerados estatisticamente significativos. Todas as análises foram realizadas usando o *software* Comprehensive Meta-Analysis versão 2.0 (Englewood, New Jersey, USA).

## RESULTADOS

Dos 4.586 estudos identificados, oito preencheram os critérios de inclusão (Figura 1). Entretanto, os dados de VO2pico de um estudo estavam na unidade de medida L.min^-1^ e outro não foi randomizado^28^^,^^29^. Após contato com os autores, eles informaram que não havia dados em mL.kg^-1^.min^-1^, unidade de medida padronizada para expressar essa variável. Portanto, seis estudos foram incluídos na análise quantitativa^30^^-^35. Desses, dois foram incluídos duplamente^30^^,^^32^, visto que preencheram os critérios de elegibilidade para duas comparações entre grupos: exercício aeróbico de moderada intensidade *versus* controle e exercício aeróbico de alta intensidade *versus* controle. Além disso, dois estudos preencheram os critérios de elegibilidade para comparação entre exercício combinado *versus* controle (treinamento aeróbico).

**Figura 1:** Fluxograma do estudo com todas etapas da revisão sistemática e metanálise.

No total, 361 participantes foram incluídos na metanálise. Desses, 155 foram incluídos no grupo exercício aeróbico (GEA), 35 no grupo exercício combinado (GEC) e 171 no grupo controle (GC). Cinquenta por cento dos estudos analisaram apenas pessoas do sexo masculino^30^^,^^33^^,^^34^, sendo que 33% analisaram pessoas de ambos os sexos e apenas um estudo (17%) não descreveu o sexo da amostra incluída. O número total de mulheres foi de 35 (9,7%).

Dois estudos (33%) apresentaram amostra composta por indivíduos sedentários e com histórico de obesidade^30^^,^^33^. Além disso, 50% apresentaram fatores de risco, como diabetes *mellitus*, hipertensão arterial sistêmica, dislipidemia e histórico de tabagismo^31^^,^^33^^-^35. Exercício aeróbico foi a modalidade prevalente entre os seis estudos (66%), sendo que, entre os quatro estudos de exercício aeróbico, dois foram adicionados duplamente, em função de apresentarem diferentes intensidades de exercício. A moderada intensidade foi utilizada em três desses quatro estudos (75%)^30^^,^^32^^,^^33^, e alta intensidade também em três estudos (75%)^30^^,^^32^^,^^35^. O exercício combinado foi utilizado em dois estudos (34%), sendo que os dois utilizaram alta intensidade para o exercício aeróbico durante o exercício combinado^31^^,^^34^.

O tempo médio de duração dos programas de intervenção foi de 10,33 ± 2,66 semanas, sendo que a frequência semanal teve duração média de 3,17 ± 0,98 e o tempo médio de duração das sessões foi de 45,83 ± 9,52 minutos. Dos seis estudos incluídos, 66% apresentaram o tempo médio após os participantes terem sofrido o IAM (22 ± 21 semanas). A Tabela 1 mostra as principais características dos estudos incluídos. A Tabela 2 traz os aspectos relacionados ao risco de viés.

**Tabela 1:** Características dos estudos incluídos para extração dos dados.

**Estudo**	**Participantes**	**Número de participantes**	**Medicamentos**	**Tempo de IAM**	**Média de Idade (anos)**	**Características Intervenção**	**Características Comparador**	**Progressão**	**Séries**	**Repetições**	**Intensidade**	**Frequência**	***Follow up***	**Duração**
				**(semanas)**										
Benetti et al., 2010	Pacientes obesos e sedentários	GAM: 29	X	GAM: 36	57,7 ± 6,1	Treinamento aeróbico de intensidade moderada e treinamento intervalado de alta intensidade	Instruídos a manter rotina normal	Não	X	X	GAM: 75% da FC máxima	5	12	60 min
		GAAI: 29		GAAI: 28							GAAI: 85% da FC máxima			
		GC: 29		GC: 36										
Moholdt et al., 2011	Pacientes com histórico de tabagismo	GAAI: 30	Betabloqueadores,	12	GAAI: 56,7 ± 10,4	Treinamento aeróbico intervalado de alta intensidade, realizado em esteira, em casa e no hospital	Treinamento aeróbico de intensidade moderada, com subida de escada e agachamentos	Não	GAAI: 4	GAAI: 4	GAAI: 85-95% da FC máxima	3	12	51 min
		GC: 59	AAS,		GC:						GC: X			
			inibidores IECA,		57,5 ± 9,3									
			estatinas,											
			antagonistas de cálcio											
			
Oliveira et al., 2014	Pacientes diabéticos, hipertensos, dislipidêmicos, obesos, tabagistas e sedentários	GAM: 47	Antiplaquetários, betabloqueadores, BCC, inibidores IECA, hipolipemiantes, diuréticos, nitrato, bloq. do recep. da angiotensina II	4	GAM: 54,8 ± 10,6	Treinamento aeróbico de intensidade moderada realizado em esteira e cicloergômetro	Foram instruídos a manter cuidados usuais	Não	X	X	70-85% da FC máxima	3	8	50 min
		GC: 37			GC:									
					58,6 ± 10,7									
**Estudo**	**Participantes**	**Número de participantes**	**Medicamentos**	**Tempo de IAM**	**Média de Idade (anos)**	**Características Intervenção**	**Características Comparador**	**Progressão**	**Séries**	**Repetições**	**Intensidade**	**Frequência**	***Follow up***	**Duração**
				**(semanas)**										
Santi et al., 2018	X	GAM: 10	X	X	55,1 ± 8,9		Cuidados usuais	Não	GAAI: 4	GAAI: 4	GAM: 60-70% da FC máxima	3	12	GAM: 40 min
		GAAI: 10				Treinamento aeróbico de intensidade moderada e treinamento intervalado de alta intensidade					GAAI: 85-95% da FC máxima			GAAI: 28 min
		GC: 10												
Khalid et al., 2019	Pacientes diabéticos e hipertensos	GTC: 26	X	X	GTC: 57,23 ± 9,75	Treinamento aeróbico intervalado + treino de força	Treinamento aeróbico intervalado	10 repetições no início depois passaram para 12 repetições treino de força	X	X	GTC: aeróbico – 60-85% FC máxima	3	6	GTC: 40 min
		GC: 26			GC:						força - X			GC: 65 min
					55,77 ± 10,45						GC: 60-85% FC máxima			
Traschel et al., 2019	Pacientes diabéticos, dislipidêmicos, hipertensos e tabagistas	GTC: 9	Aspirina, betabloqueadores, BCC, DAPT, terapia hipolipemiante, inibidores RAAS	6	GTC:	Treinamento aeróbico intervalado de alta intensidade + treino de força	Treinamento aeróbico com intensidade moderada	Não	Aeróbico:	Força:	GTC: aeróbico – 100% do pico da carga de trabalho (BORG 15)	2	12	GTC: 40 min
		GC: 10			60 ± 10				2 a 3	15 a 20	força – BORG 15			GC:
					GC:				Força: 1		GC: BORG 12 – 14			30 – 60 min
					57 ± 13									

IAM = infarto agudo do miocárdio; GAM = grupo aeróbico moderado; GAAI = grupo aeróbico alta intensidade; GTC = grupo treinamento combinado; GC = grupo controle; FC = frequência cardíaca; FC máxima = frequência cardíaca máxima; BORG = escala de sensação subjetiva ao esforço; AAS = ácido acetilsalicílico; IECA= inibidores da enzima conversora da angiotensina; RAAS = inibidores do sistema renina-angiotensina-aldosterona; BCC = bloqueadores dos canais de cálcio; DAPT = dupla antiagregação plaquetária.

**Tabela 2:** Extração do risco de viés.

**Estudo**	**Geração da sequência de randomização**	**Sigilo de alocação**	**Cegamento paciente e terapeuta**	**Cegamento avaliadores desfechos**	**Descrição perdas e exclusões**	**Dados de resultados incompletos**
Benetti et al., 2010	Baixo	*Unclear*	*Unclear*	*Unclear*	Baixo	*Unclear*
Moholdt et al., 2011	Baixo	Baixo	Baixo	Baixo	Baixo	Baixo
Oliveira et al., 2014	Baixo	Baixo	Alto	Baixo	Baixo	Baixo
Santi et al., 2018	Baixo	*Unclear*	*Unclear*	*Unclear*	*Unclear*	*Unclear*
Khalid et al., 2019	Baixo	*Unclear*	Alto	Baixo	Baixo	Alto
Traschel et al., 2019	Baixo	*Unclear*	Alto	Baixo	Alto	*Unclear*

Baixo = apresentou o critério, considerado baixo risco de viés; Alto = não apresentou o critério; *Unclear* = não está claro se possui ou não o critério.

## EFEITOS DAS INTERVENÇÕES

### Exercício Aeróbico *vs.* Controle (com e sem intervenção)

Um total de 290 participantes foram avaliados (Figura 2), 155 para o GEA e 135 para o GC. O exercício aeróbico foi associado a um aumento médio de 6,07 mL.kg^-1^.min^-1^ (IC95% 1,27 a 10,86; *I^2^*: 88%) quando comparado ao GC (p = 0,013).

**Figura 2:** Diferenças médias padronizadas no consumo de oxigênio de pico (mL.kg^-1^.min^-1^) observadas entre exercício aeróbico e grupo controle.

**Nota:** Quadrado preenchido = estimativa dos estudos específicos; Diamante preenchido = estimativas combinadas de metanálises de efeitos aleatórios; Diferença padronizada; Intervalo de confiança.

### Exercício Combinado *vs.* Controle (sem intervenção)

Setenta e um participantes foram avaliados (Figura 3). Desses, 35 eram do GEC e 36 eram do GC. O exercício combinado não apresentou diferença estatisticamente significativa quando comparado ao GC. No entanto, apresentou aumento médio de 1,84 mL.kg^-1^.min^-1^ (IC95% -1,73 a 5,42; *I^2^*: 0%) quando comparado ao GC (p = 0,312).

**Figura 3:** Diferenças médias padronizadas no consumo de oxigênio de pico (mL.kg^-1^.min^-1^) observadas entre exercício combinado e grupo controle.

**Nota:** Quadrado preenchido = estimativa dos estudos específicos; Diamante preenchido = estimativas combinadas de metanálises de efeitos aleatórios; Diferença padronizada; Intervalo de confiança.

## DISCUSSÃO

O presente estudo conduziu uma metanálise para investigar os efeitos de diferentes tipos de treinamento físico sobre o VO2pico de indivíduos que sofreram IAM. Seu principal resultado é que o exercício aeróbico foi associado a um aumento médio de 6 mL.kg^-1^.min^-1^ no VO2pico em comparação a um GC, com diferença estatisticamente significativa (p = 0,013).

Apesar da presente revisão sistemática investigar os efeitos de diferentes tipos de treinamento físico, é importante destacar a escassez de estudos que investigam os efeitos do exercício combinado sobre o VO2pico em indivíduos que sofreram IAM^31^^,^^34^ e a inexistência de estudos que investigam os efeitos do exercício de força sobre o VO2pico. Em contrapartida, o exercício aeróbico foi a modalidade mais investigada nos estudos (66%)^30^^,^^32^^,^^33^^,^^35^. Além disso, o exercício aeróbico de alta intensidade foi utilizado como GC nos dois estudos sobre exercício combinado. Portanto, nossos achados corroboram a literatura que indica o exercício aeróbico como a modalidade mais utilizada para reabilitação de indivíduos que sofreram IAM^19^^,^^20^.

Dos 361 participantes incluídos na presente metanálise, 296 eram do sexo masculino, dado que reforça a prevalência de IAM em indivíduos do sexo masculino^4^. Assim, é importante destacar que um dos fatores de risco para desenvolvimento da doença é o sexo, além da faixa etária^5^. O principal aspecto fisiológico de proteção entre o sexo feminino é o estrógeno, hormônio diretamente relacionado à proteção cardiovascular^36^. Além disso, o IAM afeta pessoas a partir dos 40-45 anos. Nessa faixa etária, os indivíduos do sexo feminino têm sua produção de estrogênio preservada. A maior incidência de IAM no sexo feminino é após a menopausa, quando ocorre uma importante redução na produção de estrogênio^36^.

O exercício aeróbico tem se mostrado eficaz na redução dos fatores de risco, assim como no aumento da capacidade funcional em indivíduos de meia idade ou idosos nas diferentes DCVs^22^^,^^37^. Programas de treinamento físico que contemplem o exercício aeróbico são importantes para promover adaptações positivas no VO2pico em indivíduos que sofreram IAM. Entretanto, é fundamental o manejo da intensidade de exercício para que se obtenha os resultados esperados durante uma intervenção^38^. O exercício aeróbico com alta intensidade parece ser o que apresenta maior magnitude de aumento no VO2pico (14,7 mL.kg^-1^.min^-1^)^30^. A alta intensidade, 85-95% do VO2pico, além de apresentar boa aderência em programas de intervenção com exercícios físicos, apresenta aumento na capacidade oxidativa dos músculos e capacidade de transporte de glicose, consequentemente melhorando a sensibilidade à insulina e o controle glicêmico^39^. O principal mecanismo de adaptação em função da alta intensidade está relacionado ao receptor-y coativador-α de peroxissoma, o mais importante regulador da biogênese mitocondrial no músculo^40^.

O mecanismo regulador do exercício aeróbico de moderada intensidade parece ser semelhante. O aspecto central é a biogênese mitocondrial, importante organela celular responsável pela atividade oxidativa dos músculos^41^^,^^42^. Aparentemente, seis 6 semanas de exercício aeróbico de moderada intensidade são necessárias para promover aumento tanto no tamanho quanto no número de mitocôndrias, gerando maior capacidade de ressíntese de adenosina trifosfato. Esse dado corrobora um dos critérios de inclusão do presente estudo, visto que foi determinado um tempo mínimo de 6 semanas de intervenção^43^. Além disso, o exercício aeróbico de modo geral aumenta a diferença arteriovenosa de oxigênio, a qual está diretamente relacionada ao aumento no VO2pico, por meio de uma maior oferta de oxigênio periférico, em função da produção de catecolaminas e maior biodisponibilidade de óxido nítrico^44^^,^^45^. A função cardíaca também apresenta maior desempenho após uma intervenção com exercício aeróbico, quando ocorre um aumento no enchimento diastólico, com uma concomitante combinação de pré-carga aumentada e relaxamento do miocárdio otimizado^46^. A melhora na função cardíaca promoverá importantes benefícios para a redução no tempo de diástole e maior eficiência no enchimento diastólico^46^.

Do ponto de vista clínico, o principal achado do presente estudo corrobora a literatura sobre o papel da reabilitação por meio de exercícios aeróbicos em indivíduos que sofreram IAM, utilizando como preferência a moderada intensidade^47^, em virtude de as diferentes diretrizes possuírem recomendação I com nível de evidência A, ou seja, altamente indicados para diferentes DCVs^19^^-^21. Um aumento médio de 6 mL.kg^-1^.min^-1^ no VO2pico, achado principal deste estudo, demonstra que o exercício físico exerce papel fundamental na redução do risco de mortalidade por DCVs, uma vez que o aumento de uma unidade no VO2pico representa redução do risco de mortalidade por DCVs de 10%^16^^,^^17^. Além disso, uma diferença de 6 mL.kg^-1^.min^-1^ pode aumentar a autonomia do indivíduo nas suas atividades diárias, visto que essa diferença pode representar uma modificação na classe funcional, tanto do New York Heart Association (NYHA) quanto Weber, quando, por exemplo, um indivíduo classe III do NYHA e classe C de Weber pode mudar para classe I do NYHA e classe A de Weber^47^^,^^48^. Portanto, o achado deste estudo é extremamente relevante para a área da reabilitação cardiovascular.

Apesar do exercício combinado não apresentar diferenças significativas em comparação a um GC (p = 0,312), é preciso destacar a inclusão de apenas dois estudos para esse tipo de intervenção, o que pode ser extremamente relevante para não encontrar significância. A diferença média de aumento no VO2pico para o exercício combinado em comparação ao GC foi de 1,84 mL.kg^-1^.min^-1^. A combinação entre exercício aeróbico e exercício de força na mesma sessão parece ser uma estratégia promissora – além de promover melhora no VO2pico, diferentes estudos apontam a importância do exercício combinado para a melhora de aspectos neuromusculares relacionados ao equilíbrio e força muscular, principalmente por meio da realização de exercício de força^23^^,^^29^. Ademais, o exercício de força promove aumento no tamanho das fibras musculares, com consequente aumento no número de mitocôndrias, facilitando a atividade oxidativa muscular^49^. Por isso, é importante que novas investigações abordem diferentes programas de treinamento físico sobre diferentes desfechos em indivíduos que sofreram IAM.

Não podemos deixar de destacar a extensa busca na literatura por artigos científicos com alta qualidade metodológica e melhor nível de evidência disponível. Mais de 4.000 estudos foram selecionados para revisão sistemática, com a leitura de títulos e resumos. Entendemos que essa é a primeira metanálise a investigar os efeitos de diferentes modalidades de treinamento físico sobre o VO2pico em indivíduos que sofreram IAM.

## CONCLUSÃO

Nossos dados demonstraram que os participantes que foram treinados com a modalidade exercício aeróbico obtiveram um aumento significativo no VO2pico. Além disso, é a modalidade mais utilizada nos estudos incluídos na presente metanálise. O exercício combinado não apresenta um aumento significativo no VO2pico, mas sua utilização em um programa de reabilitação cardiovascular é de extrema importância, visto que indivíduos que sofreram IAM, além de apresentarem redução em sua capacidade funcional, apresentam perda de força muscular e redução no equilíbrio. Portanto, é fundamental incentivar a realização de programas de treinamento físico para população que sofreu IAM, com adequado controle de intensidade e volume de exercício.
